# A New Class of Pathogenic Non-Coding Variants in GLA

**DOI:** 10.3390/ijms27020945

**Published:** 2026-01-18

**Authors:** Yujing Yuan, Xinyu Zhang, Chen Ling, Yawen Zhao, Meng Yu, Zhaoxia Wang, Yun Yuan, Zhiying Xie, Wei Zhang

**Affiliations:** 1 Department of Neurology, Peking University First Hospital, Beijing 100034, China; 2 Beijing Key Laboratory of Neurovascular Diseases, Beijing 100034, China; 3 Rare Disease Medical Center, Peking University First Hospital, Beijing 100034, China

**Keywords:** fabry disease, *GLA*, long-read sequencing, non-coding variants, sanger sequencing

## Abstract

Fabry disease (FD) exhibits a spectrum of clinical manifestations ranging from mild to severe, posing a diagnostic challenge, particularly in non-classic subtypes. Genetic testing remains a gold standard for a precise diagnosis of FD and is pivotal in genetic counseling. Although conventional approaches such as Sanger sequencing and short-read next-generation sequencing (NGS) have been successfully used to diagnose FD, they often fail to detect deep intronic variants, complex rearrangements, or large deletions or duplications. In contrast, long-read sequencing (LRS) enables comprehensive coverage of intronic and repetitive regions, facilitating precise identification of atypical variants missed by conventional methods. This case series reports two unrelated male patients with clinical, enzymatic, and pathological features consistent with FD, who tested negative for pathogenic variants in the alpha-galactosidase A (*GLA*) via Sanger sequencing and NGS. LRS identified novel non-coding variants in both patients. Patient 1 carried a ~1.7 kb insertion within intron 4, corresponding to part of a long interspersed nuclear element-1, while RNA sequencing revealed two new *GLA* transcripts. Patient 2 harbored a ~2.5 kb insertion within a SINE-VNTR-Alu retroposon element located in the 5′-untranslated region, with quantitative real-time PCR showing significantly reduced expression of normal *GLA* transcripts. These findings reveal non-coding variants that contribute to the missing heritability in FD, highlight this genomic region as a priority for future investigation, and demonstrate the potential utility of LRS in diagnostic workflows for unresolved FD cases.

## 1. Introduction

Fabry disease (FD; OMIM 301500) is a rare X-linked lysosomal storage disease caused by deleterious variants in *GLA* (NCBI, GRCh37.p13, NC_000023.10), which encodes the enzyme alpha-galactosidase A (α-Gal A; EC 3.2.1.22) [1]. This leads to accumulation of its substrate, globotriaosylceramide (Gb3), its deacylated metabolite globotriaosylsphingosine (lyso-Gb3), and related analogs [1,2]. Progressive accumulation of Gb3 and lyso-Gb3 within lysosomes across a variety of cell types and organs disrupts cellular dysfunction, leading to serious complications such as renal failure, heart failure, and stroke, and ultimately reduces life expectancy [3,4].

While α-Gal A activity testing alone is sufficient to diagnose FD in males, it is necessary to identify a pathogenic variant in *GLA* in females, who typically exhibit normal or highly variable plasma α-Gal A activity due to skewed X chromosome inactivation [5]. Routine methods for *GLA* genotyping include real-time PCR [6], multiplex PCR [7], Sanger sequencing [8,9], next-generation sequencing (NGS) [9,10], and multiple ligation-dependent probe amplification [11], which collectively identify pathogenic variants in over 96% of FD cases [12]. To date, over 1000 variants of *GLA* have been registered in the Human Gene Mutation Database (HGMD) Professional [13,14]. These include missense variants (58.5%), nonsense variants (16.5%), deletions (13.0%), splicing variants (10.5%); insertions (1.0%), and insertion-deletion (indel) (0.5%) [12]. With advances in genetic variant-detection technologies, previously undetectable intronic variants are increasingly being recognized. Compared with Sanger sequencing, long-read sequencing (LRS) offers superior detection of almost all types of variants, including deep intronic variants [15].

In 2002, Ishii et al. uncovered IVS4+919G>A, a variant that lies deep within intron 4 of *GLA* and causes aberrant splicing of *GLA* mRNA [16]. Since then, intronic variants have emerged as important contributors to the spectrum of pathogenic variants in FD. In this study, we report two novel deep intronic *GLA* variants identified by LRS in two unrelated male FD patients. These variants were not detected by classical Sanger sequencing. Bioinformatic analyses supported the pathogenic potential of both variants. These findings highlight the value of LRS in expanding molecular diagnostics when conventional approaches fall short.

## 2. Case Description

Both presented with clinical, enzymatic, and pathological features suggestive of FD, but Sanger sequencing targeting *GLA* exons and exon boundaries (National Center for Biotechnology Information reference sequence NM_000169.3) failed to detect pathogenic variants [17]. For further precise the identification and validation of the possible inversion, we performed *GLA* LRS, RNA Sequencing (RNA-seq) and quantitative reverse transcription-polymerase chain reaction (RT-qPCR). Peripheral blood was collected from patients into EDTA anticoagulated tubes. LRS was performed for the two cases using the PacBio Sequel II platform (Pacific Biosciences, Menlo Park, CA, USA) with the SMRTbell Express Template Preparation Kit 2.0 (Pacific Biosciences, Menlo Park, CA, USA). Sequencing data were processed and aligned to the human reference genome (GRCh37) using SMRT Link software (version 13.1; PacBio, Menlo Park, CA, USA) for variant calling. RNA-seq was conducted on the HiSeq 2500 or HiSeq X Ten platforms (Illumina, San Diego, CA, USA), generating 125 bp or 150 bp paired-end reads. RT-qPCR was performed using the ChamQ Universal SYBR qPCR Master Mix (Vazyme, Nanjing, China; Cat. No. Q711-03) on a 7500 Real-Time PCR System (Thermo Fisher Scientific, Waltham, MA, USA). Detailed protocols for nucleic acid extraction, library preparation, quality control, and bioinformatic analyses are provided in Supplementary File S1. Patient data were obtained through retrospective clinical review, and α-Gal A activity and lyso-Gb3 levels were determined using dried blood spot samples.

### 2.1. Patient 1

**Figure 1:**
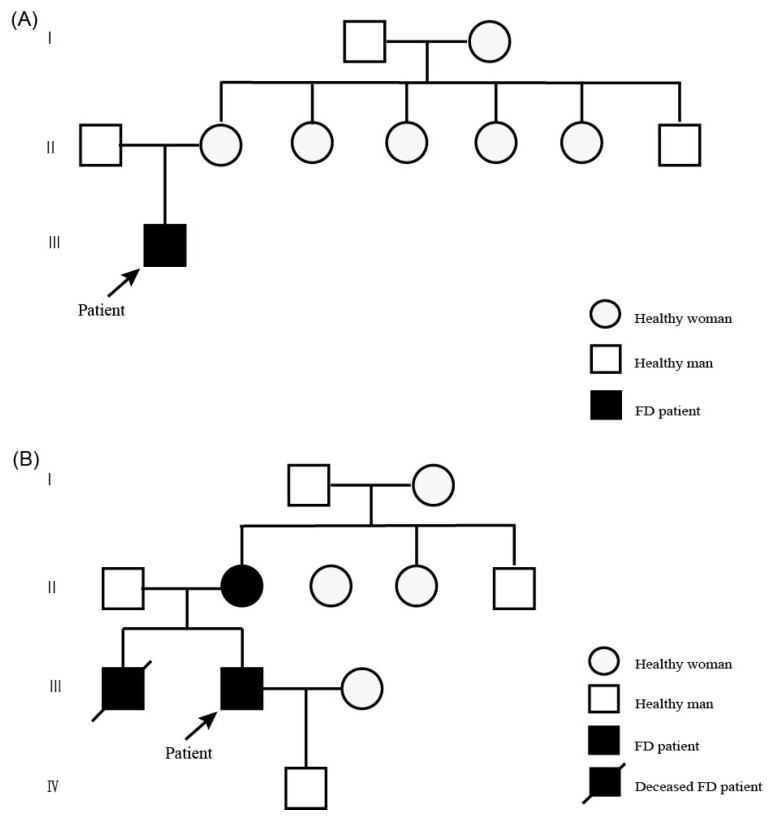
Family pedigrees of the two case patients. (**A**,**B**) Family pedigrees of Patient 1 (**A**) and Patient 2 (**B**). Arrows indicate probands. Squares represent males; circles represent females. Solid black symbols indicate individuals affected by Fabry disease (FD); black symbols with a slash indicate deceased individuals affected by FD.

**Figure 2:**
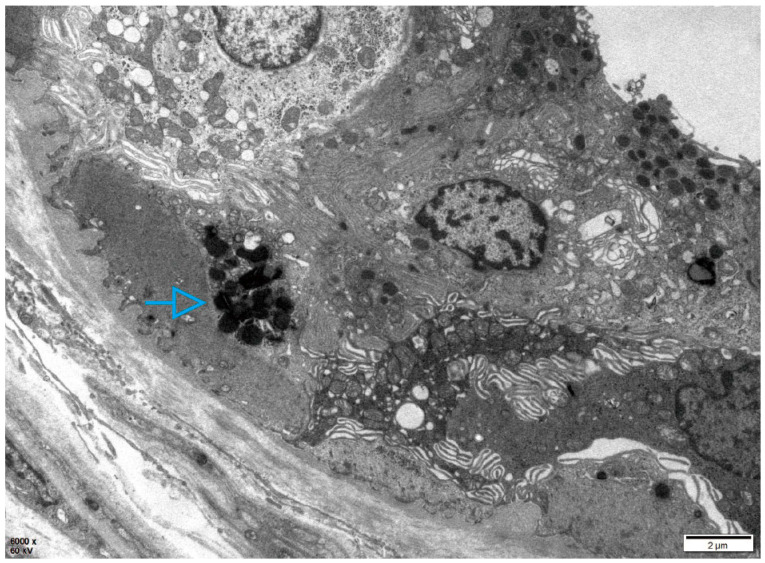
Electron microscopy of the skin biopsy in Patient 1. Electron microscopic images of the skin biopsy, revealing myeloid bodies (blue arrows) within epithelial cells of sweat glands.

Patient 1, a 20-year-old Han Chinese man, presented with hypohidrosis and unexplained acroparesthesia since childhood, triggered by physical exertion, heat, or emotional stress. Additional symptoms included memory decline, occasional palpitations, dizziness, intermittent tinnitus, fatigue, and reduced exercise tolerance. The patient had undergone intussusception surgery at 8 months of age. Family history was negative for FD (Figure 1A). Renal, heart, and brain assessments were unremarkable. Biochemical testing revealed markedly reduced α-Gal A enzyme activity in leukocytes (0.64 µmol/L/h; reference range: 2.40–17.65) and elevated plasma lyso-Gb3 levels (19.84 ng/mL; reference: <1.11). Skin biopsy showed myeloid bodies in epithelial cells of sweat glands, consistent with FD (Figure 2). Sanger sequencing did not detect pathogenic *GLA* variants.

**Figure 3:**
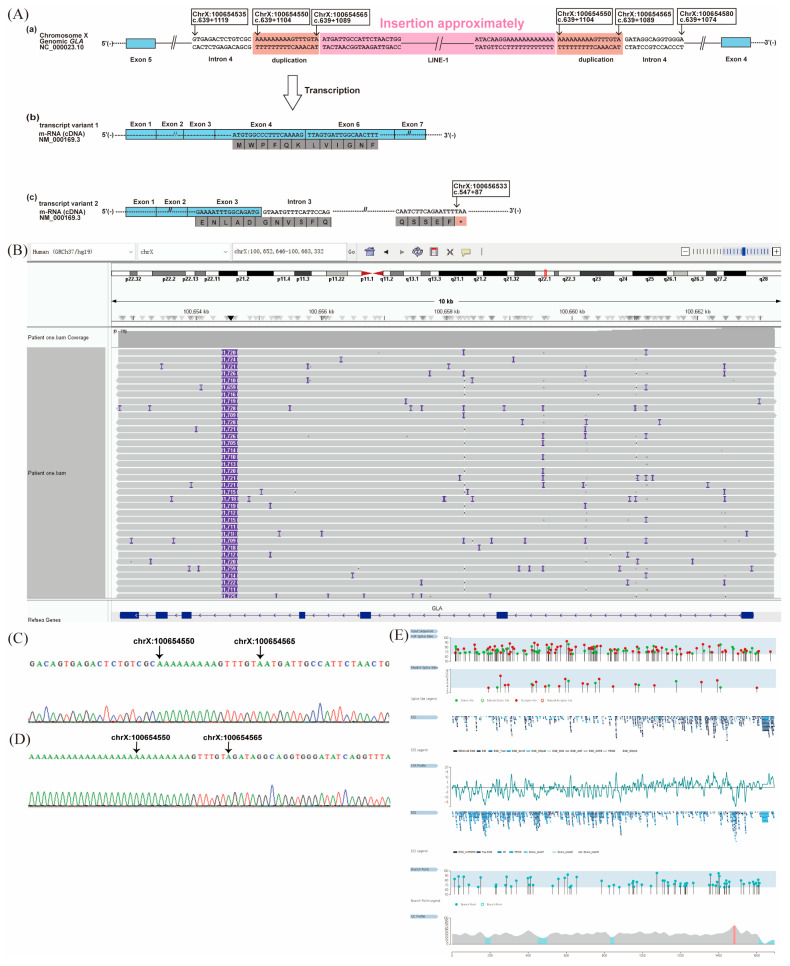
Novel deep intronic structural variant in *GLA* and its impact on aberrant splicing in Patient 1. (**A**) Schematic diagram of the abnormal structural variant of the *GLA* intron 4. (**a**) Schematic of abnormal splicing caused by a ~1.7 kb insertion in *GLA* intron 4, flanked by a 16 bp non-tandem duplication and mostly annotated as a long interspersed nuclear element-1 (LINE-1). Each Exon is shown as a light blue box. Pink represents the insertion sequences. (**b**) Transcript variant 1 showing exon 5 skipping, leading to an in-frame deletion. (**c**) Transcript variant 2 showing intron 3 retention, causing a frameshift and premature stop codon. (**B**) Integrative Genomics Viewer screenshot from long-read sequencing confirming the ~1.7 kb insertion in intron 4. (**C**,**D**) Sanger sequencing confirming the 5′ and 3′ breakpoints. (**E**) Genomic positions of the natural and cryptic donor (green) and acceptor (red) splice sites within the inserted as predicted by Human Splicing Finder. The exonic splicing regulatory (ESR) ratio (ESEs/ESSs) along the same genomic region. Position of the natural and alternative branch points (light blue) along the analyzed genomic region. * Indicates stop codon.

RNA-seq of the case patient revealed two aberrant *GLA* transcripts: one exhibited exon 5 skipping (NM_000169.3:r.640_801del), resulting in an in-frame deletion; the other showed intron 3 retention (NM_000169.3:r.547_548ins547+1_547+89), introducing a premature termination codon (NP_000160.1:p.Gly183fsTer28; Figure 3A). The latter transcript was predicted to undergo nonsense-mediated decay.

LRS identified a ~1.7 kb insertion within intron 4 of *GLA*, located between g.100654565 and g.100654566 (NC_000023.10). The insertion corresponded to part of a long interspersed nuclear element-1 (LINE-1), annotated using the RepeatMasker web server under default settings (https://www.repeatmasker.org/cgi-bin/WEBRepeatMasker, accessed on 5 June 2024). A 16 bp non-tandem duplication from intron 4 was present at both ends of the insertion. According to Human Genome Variation Society nomenclature [18], this variant is described as NC_000023.10: g.[100654565_100654566ins1.7kb;100654550_100654565dup] (Figure 3A,B; see Supplementary File S2 for the insert sequence). Sanger sequencing confirmed 5′ and 3′ breakpoints (Figure 3C,D), and maternal testing was negative for the insertion.

Splicing analysis of the 1.7 kb insert using the Human Splicing Finder (HSF) web tool (https://hsf.genomnis.com) identified 152 splicing signals, including 85 acceptor and 67 donor splice sites, 1134 exonic splicing silencer (ESS) sites, 987 exonic splicing enhancer (ESE) sites, and 74 branch point sequences (BPSs). The exonic splicing regulatory (ESR) ratio, calculated as the number of ESEs divided by the number of ESSs, was <1 (1134/987) (Figure 3E).

### 2.2. Patient 2

Patient 2, a 37-year-old Han Chinese man, presented with acroparesthesia and hypohidrosis starting at the age of 8 years. His medical history included hypertension, nephrotic syndrome, and a positive family history of FD. He underwent dialysis for 4 years prior to undergoing a kidney transplant at the age of 35 years. During the course of his illness, he also experienced tinnitus, hearing loss, palpitations, and diarrhea. Echocardiography showed left ventricular hypertrophy (left ventricular posterior wall thickness: 1.6 cm). Lab results revealed reduced α-Gal A activity (0.49 µmol/L/h), and elevated lyso-Gb3 levels (52.01 ng/mL). His older brother developed similar symptoms at the age of 8 years and had a history of hypertension, hypertrophic cardiomyopathy, and lacunar infarction; he died after initiating dialysis at the age of 33 years. The patient’s 56-year-old mother reported burning sensations in her feet beginning at the age of 8 years, which disappeared after childbirth. The patient is currently receiving enzyme replacement therapy (Figure 1B).

**Figure 4:**
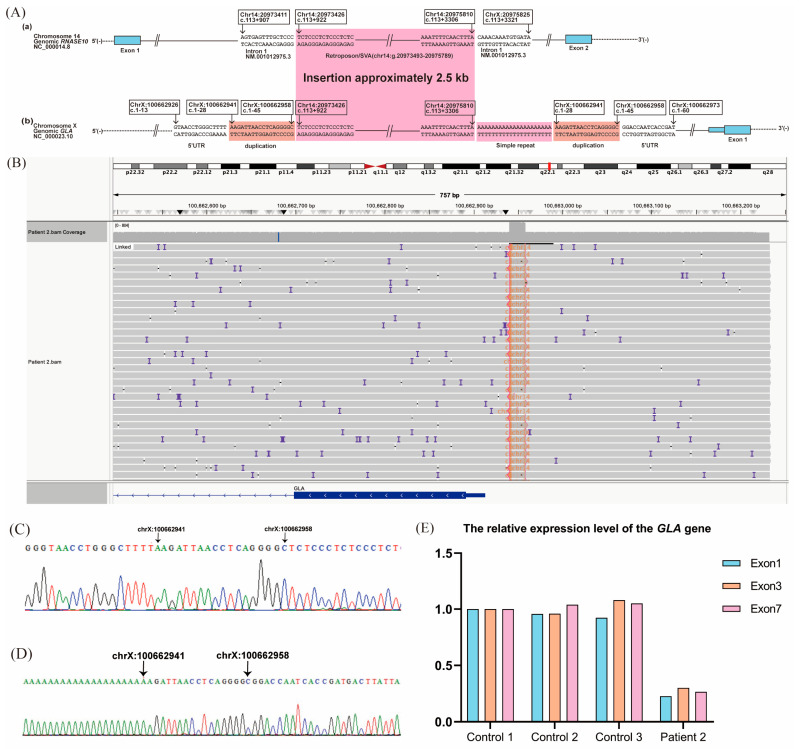
Novel structural variant in the *GLA* 5′-untranslated region (UTR) and its transcriptional impact in Patient 2. (**A**) Schematic diagram of the abnormal structural variant of the *GLA* 5′-UTR. (**a**,**b**) Structural variant in the 5′-UTR, with a ~2.5 kb insertion sequence flanked by a 21-nucleotide adenine repeat and an 18 bp deletion (g.100662941_100662958dup). The inserted sequence shows homology to a deep intronic region of *RNASE10* intron 1 and includes a SINE-VNTR-Alu (SVA) retroposon element. Each Exon is shown as a light blue box. Pink represents the insertion sequences. (**B**) Integrative Genomics Viewer screenshot of long-read sequencing confirming the ~2.5 kb insertion. (**C**,**D**) Sanger sequencing validation of the insertion. (**E**) RT-qPCR analysis showing reduced expression of *GLA* transcripts containing exons 1, 3, and 7 of *GLA* compared with healthy controls.

Unlike Patient 1, RNA-seq did not detect aberrant *GLA* transcripts. However, RT-qPCR analysis showed reduced expression of exons 1, 3, and 7 in normal *GLA* transcripts compared with healthy controls (Figure 4E).

LRS identified a 2.5 kb insertion within the 5′-untranslated region (UTR) located between g.100662958 and g.100662959, along with a 21-adenine nucleotide duplication and an 18 bp duplication (g.100662941_100662958) downstream of the insertion. RepeatMasker annotated the inserted sequence as part of a SINE-VNTR-Alu (SVA) retroposon element. Homology analysis using the Human BLAT Search tool (GRCh37/hg19) revealed similarity between the insertion sequence and a deep intronic region of *RNASE10* intron 1 (chr14:g.20973426-20975810). This variant is described as NC_000023.10: g.[100662958_100662959ins2.5kb;G[21];100662941_100662958dup] (Figure 4A–D). Sanger sequencing confirmed the variants.

## 3. Discussion

We report two novel non-coding *GLA* variants in two unrelated male patients with FD, including a 1.7 kb LINE-1 insertion within intron 4 associated with abnormal splicing, and a 2.5 kb insertion in the 5′-UTR homologous to *RNASE10* intron 1 linked to reduced expression of normal transcripts. Although NGS, which enables analysis of numerous genes and detection of complex variants, has been successfully used in FD diagnosis, deep intronic variants may still be missed because of limitations in exon-focused panels. LRS can identify complex genetic variants, including deep intronic variants and structural rearrangements, complementing routine diagnostic methods. In a cohort study of 207 Japanese patients with FD, seven (3.4%) had no pathogenic variants detected in the exonic regions or exon–intron boundaries of *GLA* using standard gene analysis [12]. In a study by Nowak et al., two of 12 patients showed no pathogenic variants on Sanger sequencing. Multiple amplicon sequencing subsequently identified a deep intronic variant (c.547+404T>G) in one patient, while mRNA and cDNA sequencing showed an exon 2 deletion in the other [19]. In a 2024 cohort of 82 FD patients, Yao et al. reported three deep intronic variants identified by long-range PCR coupled with LRS that had been missed by Sanger sequencing [15]. Similarly, our study uncovered two insertional variants, one in intron 4 and the other in the 5′-UTR of *GLA*, in FD patients who previously tested negative by conventional sequencing. Based on clinical symptoms, α-Gal A activity, and lyso-Gb3 levels, these two patients were classified as having non-classic and classic FD, respectively [17]. Although direct molecular testing was not performed for the patient 2 deceased brother or the mother, their clinical histories are highly suggestive of FD. The brother’s early-onset major organ involvement and the mother’s classic neuropathic pain, in the context of the identified *GLA* variant in the proband, strongly support the carrier status of the mother and the affected status of the brother, consistent with X-linked inheritance.

Located more than 100 bp away from exon–intron boundaries, these deep intronic variants can affect ESEs or ESSs, potentially causing misregulated splicing [20]. Splicing of pre-mRNAs removes introns and joins exons to form mature mRNA transcripts for translation. This process is guided by signals such as splice sites, BPSs, and regulatory elements, including ESEs and ESSs, which promote or inhibit the inclusion of coding segments in the final mRNA [21,22,23,24]. The prevalent c.639+919G>A pathogenic variant disrupts the binding of heterogeneous nuclear ribonucleoprotein A1/A2 to an ESS, causing pseudoexon activation and FD [25]. In our Patient 1, the ESR ratio was <1, suggesting a predominance of splicing silencers over enhancers, which may contribute to exon skipping or impaired exon recognition during pre-mRNA splicing.

LINE-1 elements can drive inherited diseases through various mechanisms, including de novo LINE-1 insertions, insertion-mediated deletions, and genomic rearrangements [26]. Wimmer et al. [27] reported a patient with a LINE-1 insertion in intron 9 of the *NF1* gene, resulting in the skipping of the preceding exon and exonization of a short portion of the LINE-1 sequence. Alesi et al. [28] described two siblings affected by neurofibromatosis type 1, in whom insertion of a 5′-truncated inverted LINE-1 element into intron 15 led to skipping of the upstream exon and generation of an alternative transcript lacking exon 15. Similarly, our Patient 1 was found to harbor a ~1.7 kb insertion derived from a LINE-1 element, predicted to disrupt normal splicing and result in aberrant transcription.

The 5′- and 3′-UTRs are critical for post-transcriptional gene regulation, influencing processes such as mRNA processing, stability, and translation initiation [29]. The 5′-UTR, plays a particularly important role in ribosome recruitment and translation efficiency, impacting cellular proteome expression [30,31]. Variants in the 5′-UTR have been shown to impair translation and lead to disease, as exemplified by the heterozygous deletion of *MEN1* [32] and a variant in *FGF13* [33]. SVA insertions can affect gene expression through mechanisms such as exon trapping and alternative splicing, potentially leading to truncated proteins [34]. Pathogenic SVA insertions have been implicated in various diseases, including X-linked dystonia-parkinsonism (XDP), breast cancer, Fukuyama muscular dystrophy, and Batten disease [34]. XDP cell models exhibit multiple transcriptional abnormalities surrounding the exons flanking the SVA insertion, including aberrant alternative splicing, partial intron retention, decreased transcription of exons, and decreased levels of the full-length *TAF1* mRNA [35]. In our Patient 2, we identified an SVA insertion within the *GLA* 5′-UTR that resulted in reduced gene expression. This discovery represents a crucial step toward understanding the molecular mechanisms by which retrotransposons contribute to FD pathogenesis, a phenomenon not previously reported.

## 4. Conclusions

Our study broadens the known genetic spectrum of *GLA* variants and provides further insight into the molecular basis of FD. When conventional Sanger sequencing fails to detect pathogenic variants, LRS should be considered. Integrating LRS into the diagnostic workflow ensures a more comprehensive approach, reducing the risk of missing clinically relevant variants in cases with negative results from conventional testing.

## Data Availability

The original contributions presented in this study are included in the article/Supplementary Materials. Further inquiries can be directed to the corresponding authors.

## Supplementary Materials

The following supporting information can be downloaded at: https://www.mdpi.com/article/10.3390/ijms27020945/s1. Ref. [36] is cited in Supplementary Materials file.
