# Supplementary material for: A New Class of Pathogenic Non-Coding Variants in GLA
Source: Int J Mol Sci. 2026 Jan 18;27(2):945. doi: 10.3390/ijms27020945 (PMC12841649; doi:10.3390/ijms27020945)
Supplement: Supplementary file 1 [file ijms-27-00945-s001.zip › Supplementary File S1.pdf]

A New Class of Pathogenic Non-Coding Variants in GLA

Yujing Yuan [1], Xinyu Zhang [1], Chen Ling [1], Yawen Zhao [1], Meng Yu [1], Zhaoxia Wang [1,2,3], Yun Yuan [1,2,3], Zhiying Xie [1,*] and Wei Zhang [1,2,3,*]

1 Department of Neurology, Peking University First Hospital, Beijing 100034, China

2 Beijing Key Laboratory of Neurovascular Diseases, Beijing 100034, China

3 Rare Disease Medical Center, Peking University First Hospital, Beijing 100034, China

* Correspondence: xiezhiyingxzy@bjmu.edu.cn (Z.X.); neurozw@163.com (W.Z.);
Tel.: +86-010-8357-2588 (W.Z.); Fax: +86-010-6655-1107 (W.Z.)

LRS, RNA-seq, and bioinformatic analyses

Sample collection and DNA extraction

Peripheral blood was collected from participants into ethylene diamine tetraacetic acid (EDTA) anticoagulated tubes. Genomic DNA was extracted using the Blood DNA Extraction Kit (Magigene, Guangzhou, China; Cat. No. IVD3102-F-96), following the manufacturer's instructions. LRS was performed on the two case patients using the PacBio Sequel II platform (Pacific Biosciences, Menlo Park, CA, USA), as previously described[1]. Briefly, target read lengths ranged from 2,556 to 3,154 bp, with 1× coverage across the target region reaching 97.00% to 99.60%. Libraries were prepared using the SMRTbell Express Template Preparation Kit 2.0 (Pacific Biosciences, Menlo Park, CA, USA), following the manufacturer's protocol. Raw data were processed for quality control, demultiplexing, and variant calling using SMRT Link software (version 13.1;

PacBio, Menlo Park, CA, USA). Subreads were aligned to the human reference genome (GRCh37), and novel transcript isoforms were visualized using Integrative Genomics Viewer (IGV).

RNA sequencing (RNA-seq) and bioinformatic analysis

RNA-seq was performed in collaboration with GrandOmics. Total RNA was extracted using the RNeasy Mini Kit (Qiagen, Hilden, Germany; Cat. No. 74104), following the manufacturer's protocol. RNA integrity was assessed by agarose gel electrophoresis and quantified using a NanoDrop 2000 spectrophotometer (Thermo Fisher Scientific, Waltham, MA, USA). Libraries were constructed using the KAPA Library Preparation Kit (Illumina), following the manufacturer's instructions. Sequencing was conducted on the HiSeq 2500 or HiSeq X Ten platforms (Illumina, San Diego, CA, USA), generating 125-bp or 150-bp paired-end reads.

Quantitative real-time PCR (RT-qPCR)

Relative gene expression was measured using the ChamQ Universal SYBR qPCR Master Mix (Vazyme, Nanjing, China; Cat. No. Q711-03) on a 7500 Real-Time PCR System (Thermo Fisher Scientific, Waltham, MA, USA).

1. Deng J, Yu J, Li P, Luan X, Cao L, Zhao J, et al. Expansion of GGC Repeat in GIPC1 Is Associated with Oculopharyngodistal Myopathy. *American Journal of Human Genetics*. (2020). 106(6): p. 793-804. DOI: 10.1016/j.ajhg.2020.04.011.
