# Supplementary material for: A New Class of Pathogenic Non-Coding Variants in GLA
Source: Int J Mol Sci. 2026 Jan 18;27(2):945. doi: 10.3390/ijms27020945 (PMC12841649; doi:10.3390/ijms27020945)
Supplement: Supplementary file 1 [file ijms-27-00945-s001.zip › Supplementary File S2.pdf]

AAAAAAAAAAGTTTGTAATGATTGCCATTCTAACTGGTGTGAGATGATATCTCA
TAGTGGTTTTGATTTGCATTTCTCTGATGGCCAGTGATGATGAGCATTTCTTCAT
GTGTTTTTTTGGCTGCATAAATGTCTTCTTTTGAGAAGTGTCTGTTCATGTCCTT
CGCCCACTTTTTGATGGGGTTGTTTGTTTTTTTCTTGTAAATTTGTTTGAGTTCA
TTGTAGATTCTGGATATTAGCCCTTTGTCAGATGAGTAGGTTGCAAAAATTTTC
TTCTCCATGTTGTAGGTTGCCTGTTCACTCTGATGGTAGTTTCTTTTGCTGTGCA
GAAGCTCTTTATTAGATCATTAGATCCCATTTGTCAATTTGTCTTTTGTTGCCATT
GCTTTTGGTGTTTTGGACATGAAGTCCTTGCCCACGCCTATGTCCTGAATGGTA
ATGCCTAGGTTTTCTTCTAGGGTTTTTATGGTTTTAGGTTTAACGTTTAAATCTTT
AATCCATCTTGAATTGATTTTTGTATAAGGTGTAAGGAAGGGATTCCAGTTTCA
GCTTTCTACATATGGCTAGCCAGTTTCCCAGCACCATTTATTAAATAGGGAATCC
TTTCCCCATTGCTTGTTTTTCTCAGGTTTGTCAAAGATCAGATAGTTGTAGATAT
GCGGCATTATTTCTGAGGGCTCTGTTCTGTTCCATTGATCTATATCTCTGTTTTG
GTACCAGTACCATGCTGTTTTGGTTACTGTAGCCTTGTAGTATAGTTTGAAGTC
AGGTAGTGTGATGCCTCCAGCTTTGTTCTTTTGGCTTAGGATTGACTTGGCAAT
GCGGGCTCTTTTTTGGTTCCATATGAACTTTAAAGTAGTTTTTTCCAATTCTGTG
AAGAAAGTCATTGGTAGCTTGATGGGGATGGCATTGAATCTGTAAATTACCTTG
GGCAGTATGGCCATTTTCACGATATTGATTCTTCCTACCCATGAGCATGGAATGT
TCTAAACAACAGGTGCTGGAGAGGATGCGGAGAAATAGAACACTTTTACACT
GTTGGTGGGACTGTAAACTAGTTCAAACCATTGTGGAAGTCAGTGTGGCGATT
CCTCAGGATCTAGAACTAGAAATACCATTTGACCCAGCCATCCCATTACTGGGT
ATATACCCAAATGAGTATAAATCATGCTGCTATAAAGACACATGCACACGTATGT
TTATTGCGGCACTATTCACAATAGCAAAGACTTGGAACCAACCCAAATGTCCA
ACAATGATAGACTGGATTAAGAAAATGTGGCACATATACACCATGGAATACTAT
GCAGCCATAAAAAATGATGAGTTCATATCCTTTGTAGGGACATGGATGAAATTG
GAAACCATCATTCTCAGTAAACTATCGCAAGACAAAAAACCAAACACCGCATA
TTCTCACTCATAGGTGGGAATTGAACAATGAGATCACATGGACCCAGAAGGGG
AATATCACACTCTGGGGACTGTGGTGGGGTCGGGGGAGGGGGGAGGGATAGC
ATTGGGGAGATATACCTAATGCTAGATGACACATTAGTGGGTGCAGTGCACCAG
CATGGCACATGTATACATATGTAACTAACCTGCACAATGTGCACATGTACCCTA
AAACTTAGAGTATAATAAAAAAAAAAAAAAAAAAAAAAAAAAAAAAAAAAA
AAAAGAATTCAAAATACAAGGAAAAAAAAAAAAAAAAAAAAA
